# 

**Published:** 1879-03

**Authors:** 


### CONTENTS.

COMMUNICATIONS.

Histology of the Teeth. *By J. H. Siddall, Canton, O.* ........ 97
"Why do our Fillings Fail." *By Dr. L. D. Pearce, Iowa City* ........ 101

PROCEEDINGS OF SOCIETIES.

Proceedings of the Tennessee Dental Society ........ 112

MISCELLANEOUS.

Keratoscopy. *By Julia W. Carpenter, M. D.* ........ 119
Tumor of the Lower Jaw, the Seat of Vicarious Menstruation, Removal of one-half of the Bone, Followed by Rapid Recovery. *By Donald Maclean, M. D.* ........ 128
Tetanus ........ 131
How to Select ........ 135
Preservation of Anatomical Specimens with Paraffine ........ 136
How to Cough ........ 137

EDITORIAL.

The Northern Ohio Dental Association ........ 138
Illinois Dental Society ........ 138
California State Dental Association ........ 139
Commencement ........ 140

